# Peri-implant marginal bone topography at the uncover stage: a classification proposal and retrospective observational study

**DOI:** 10.1186/s40729-026-00675-5

**Published:** 2026-04-06

**Authors:** Karen Villarreal-Arizpe, I.-Ching Wang, Priyanka Pitchumani, Oscar Durán-Garnica, Hsun-Liang Chan

**Affiliations:** 1 Division of Periodontology, The Ohio State University College of Dentistry, Columbus, OH USA; 2 Periodontics, University of Iowa College of Dentistry, Iowa, USA

**Keywords:** Bone resorption, Dental implants, Alveolar bone loss, Implant prognosis, Wound healing, Microsurgery

## Abstract

Most studies have examined early crestal bone loss/remodeling on radiographs after the first year of loading and subsequent late bone loss. Limited research has investigated the patterns of bone remodeling in 360 degrees during the initial stages of implant healing. This study aimed to classify peri-implant marginal bone remodeling patterns at the implant uncovering stage. A total of 74 patients with 106 implants were included. At uncovering stage under an operating microscope, peri-implant bone conditions were recorded and categorized into four types: Type 1A (facial or lingual remodeling ≤ 2 mm with intact interproximal bone), Type 1B (facial or lingual remodeling ≤ 2 mm with interproximal remodeling ≤ 2 mm), Type 2 (circumferential remodeling ≤ 2 mm), and Type 3 (bone remodeling > 2 mm at any site). Results showed that 43% of implants exhibited no bones loss, while 57% presented various degree of bone remodeling, of which 58% Type 1A, 18% Type 1B, 17% Type 2, and 7% Type 3 in this specific cohort. Multinomial logistic regression identified ridge preservation history, simultaneous guided bone regeneration, and age as significant predictors of the bone remodeling variations. This new classification provides a framework for assessing early bone remodeling pattern and could serve as a foundation for future studies exploring the relationship between early remodeling, peri-implantitis risk, and long-term implant outcomes.

## Introduction

Implant stability, which depends on the degree of biological as well as biomechanical integration with the surrounding bone, is the most crucial requirement for long-term implant success [1]. Ideally, implants should be completely covered by bone with healthy and adequate soft tissue seal to resist bacterial as well as other mechanical insults. Nevertheless, early crestal/marginal bone remodeling is common and often referred as physiological bone remodeling [1, 2]. Historically, a smooth-surfaced and external-hexed implant has been considered as successful if bone loss was ≤ 2 mm during the first year of loading and < 0.2 mm annually thereafter [3, 4]. Recent proposals suggest different thresholds, such as < 1.5 mm [5], 1.5–2 mm [6], or fewer than 3 threads [7, 8]. Modern success criteria also include peri-implant health, prosthetic outcomes, and patient satisfaction, though osseointegrations remains the predominant success criterion in implantology [5].

Many factors contribute to the observed early bone remodeling, including soft tissue thickness and formation of the biologic width [9, 10], bacterial leakage from the microgap [2], spontaneous early implant exposure [11], repeated disconnection of prosthetic components [12], implant-abutment connection designs [13], vertical interface location [14], occlusal overload [8, 15], abutment height [16], implant position [17], and surgical trauma [18, 19]. Although bone remodeling is mostly identified between uncover and the 1st year of function, it may also occur during submerged healing [20]. Little is known about early bone remodeling in submerged, non-loaded implants [21]. Proposed causes include thin bone [22], microfractures [23], overheating [2], saliva contamination within the cover screw [24, 25], within others.

Most studies measure bone remodeling at or after prosthesis delivery [17, 26]. Fernández-Formoso et al. reported a mean bone loss of 0.72 mm at restoration cementation [27]. Kütan et al. found 0–1.1 mm in equicrestal implants and 1–1.2 mm in subcrestal placements [28]. In a preclinical study, Ericsson et al. found 1.3 mm bone loss before uncovering [29]. Saleh et al. meta-analysis found bone remodeling of 0.03 mm, 0.57 mm and 0.52 mm when the implant was placed supracrestal, equicrestal, and subcrestal, respectively [17].

On the other hand, biological complications such as peri-implant mucositis and peri-implantitis are significant concerns linked to bacterial challenge [30]. Schwartz et al. described defect morphology, finding circumferential defects most common (55.3%) [31]. García-García also identified circumferential defects as the most prevalent (32.6%) [32], while Monje reported the infraosseous 2–3 walls defects (55%) as the most common [22]. Such classifications help treatment decisions. However, no current system exists for early peri-implant bone loss to guide follow-up, restoration timing, or intervention strategies.

The relationship between early bone remodeling and late pathological bone loss around dental implants remains under researched. Early bone remodeling/loss and the resulting exposure of the implant surface have been proposed as potential risk factors for peri-implantitis [33]. In effect, Song et al. showed that implants with buccal dehiscence defects are more susceptible to progressive bone loss when exposed to plaque [34]. Additionally, in a 10-year study, Windael et al. found that early bone loss > 0.5 mm significantly increased peri-implantitis risk and implant failure [35]. Thus, a classification used at implant uncover phase may help correlate remodeling patterns with long-term outcomes, supporting preventive strategies and implant longevity.

Given the research gap that studies on bone morphology at uncovering are fewer compared to those on marginal bone resorption after prosthetic placement, it is the 1st step to characterize the early bone loss pattens. Therefore, the primary aim of this present study is to propose a classification of early bone remodeling at the uncover stage and evaluate its distribution in a retrospective human cohort.

## Materials and methods

This study adhered to the STROBE [36] guidelines.

### Compliance of clinical study regulations

Approval for this study was obtained from the Office of Responsible Research Practices at The Ohio State University (IRB 2024H0373) and conducted in accordance with the guidelines and the Declaration of Helsinki, as revised in 2013.

### Patient selection

Electronic patient records were reviewed of the patients receiving at least one implant placement surgery during Feb 2021 and Aug 2023 by single periodontist with 17 years of surgical experience (HLC) in 3 private practices. All surgeries, including implant placement, bone augmentation, and 2nd stage, were performed under an operating microscope (Pico, Zeiss, Oberkochen, Germany) Inclusion criteria were as follows: Patients with systemic health or controlled mild/moderate systemic diseases corresponding to the American Society of Anesthesiologists (ASA) Class 1 or 2; patients with oral health without active periodontal inflammation or any dental diseases; only non-smokers, former smokers quitting smoking more than 3 months, or light smokers (less than 10 cigarettes/day) were included for implant surgery; patients may receive prior alveolar ridge preservation, prior staged guided bone regeneration/or sinus augmentation, simultaneous guided bone regeneration/or sinus augmentation; and video recordings from the operating microscope during the 2nd stage uncover stage were available to classify the peri-implant bone topography. Exclusion criteria: video recordings at the 2nd stage when not available.

### Surgery protocol

The implants of selection in this study were Zimvie TSVM (conical implant with internal hex connection and with a 1.8 mm coronal micro thread in the coronal part and a 0.5 mm machined collar; Zimmer Dental Inc., Carlsbad, CA, USA), placed with a delayed time protocol (> 6 months after tooth extraction) in the maxilla and/or mandible at the equicrestal level after elevating a full thickness flap [37]. Osteotomy sequences followed the manufacturer’s instructions. Implant length was chosen according to the available ridge height or expected ridge height in cases of simultaneous ridge augmentation. The selection of the implant diameter largely depended on the replacing tooth, i.e., maxillary centrals 3.7/4.1 mm, maxillary laterals/mandibular incisors: 3.1 mm, premolars 3.7 mm, molars 4.7 mm. After the initial submerged healing period of 3–17 months, the second stage was performed with either a punch technique when adequate keratinized mucosa is available and minimal tissue trauma is indicated, a crestal incision and full thickness flap primarily in the posterior region, or an apically positioned flap for increasing keratinized mucosa width.

### Defect and patient assessment

At the time of the second stage surgery, the surrounding hard tissue was carefully assessed and classified. Configuration and defect depth of the peri-implant bone loss were recorded to the nearest 1 mm by a calibrated examiner (HC) using a calibrated periodontal probe (University of North Carolina [UNC] Probe, Hu-Friedy, Chicago, IL, USA) under the operating microscope (range of magnification was from ~ 5 to 25).

**Fig. 1:**
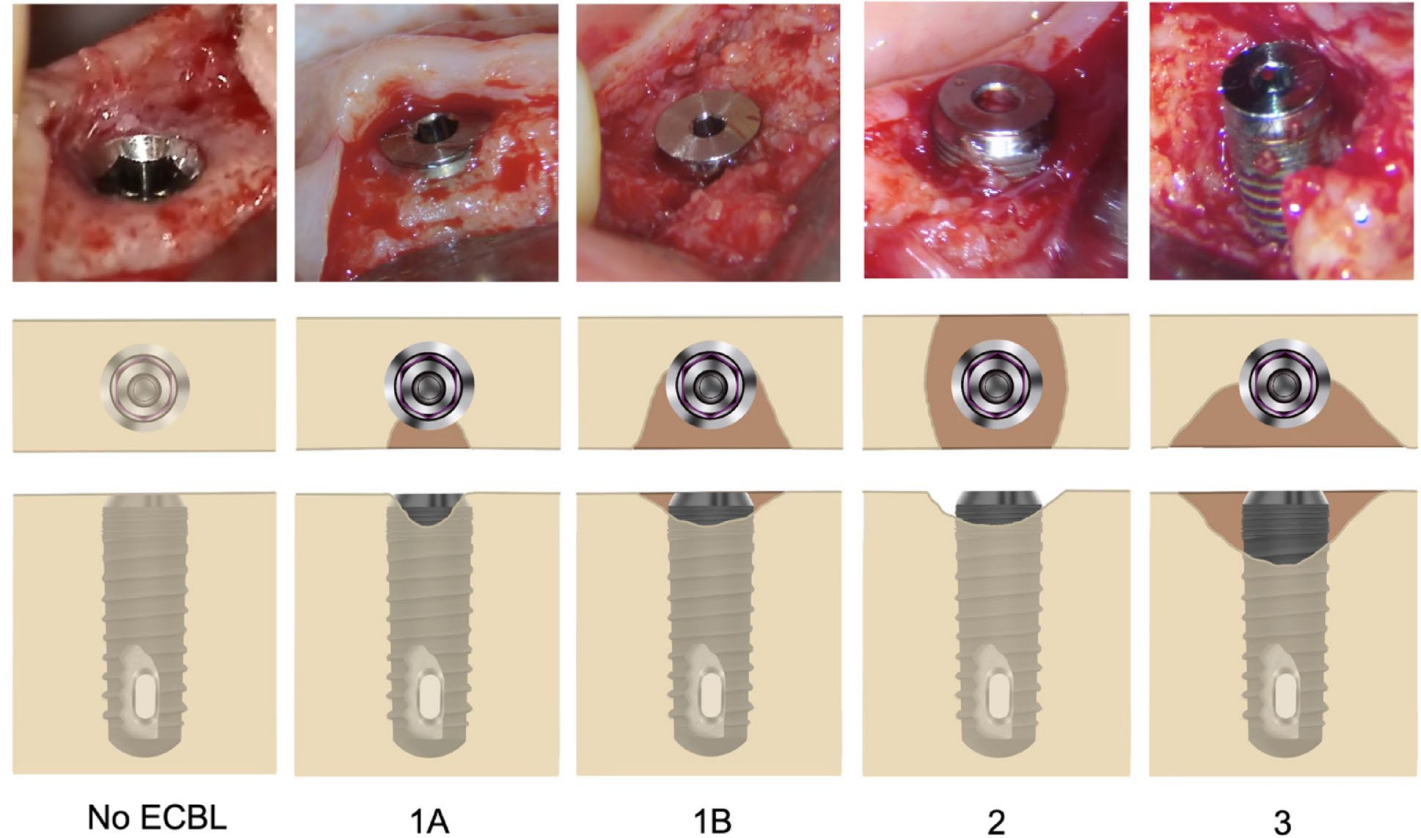
Peri-implant early crestal bone loss classification (ECBL: early crestal bone loss)

The defects were classified as follow (Table 1 and Fig. 1):

- Type 1A: Facial or lingual/palatal bone remodeling with implant exposure up to 2 mm, with intact interproximal bone
- Type 1B: Facial or lingual/palatal bone remodeling with implant exposure up to 2 mm, combined with interproximal bone remodeling up to 2 mm
- Type 2: Circumferential bone loss up to 2 mm, including facial, interproximal, and lingual/palatal crestal bone
- Type 3: Crestal bone loss more than 2 mm in any of the sites

**Table 1:**
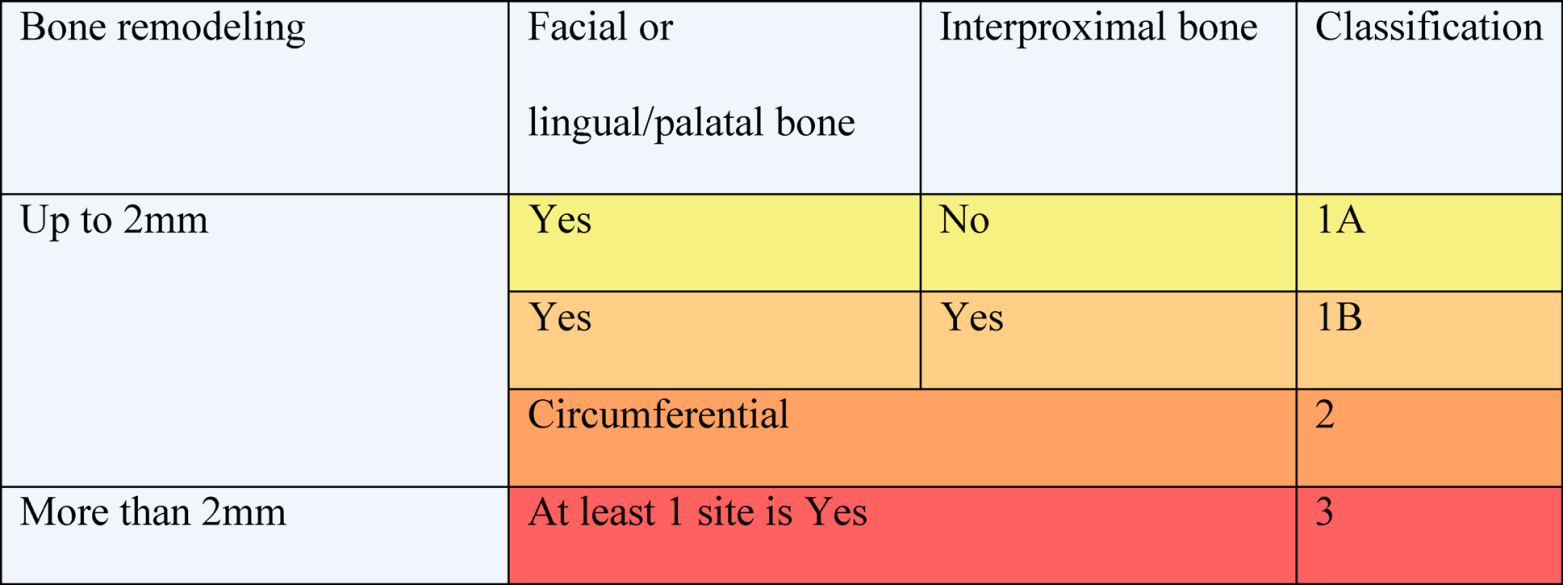
Peri-implant early crestal bone loss classification

Additional patient- and implant-related factors were documented, including age at the time of the treatment, gender, total number of implants, implant position (mandibular anterior, mandibular posterior, maxillary anterior, maxillary posterior), smoking status, former smoker, and nonsmoker, diabetes, and other relevant medical history.

### Statistical analysis

The data collected was documented in an Excel spreadsheet (Mac OS Microsoft® Excel v.16.52), and a percentage graph was subsequently created to illustrate the frequency of each defect as a total of uncovered implants and the frequency as a total of the defects. The rest of the data was summarized in tables, and the percentages were calculated with excel formulas.

Descriptive statistics with mean ± standard deviation (minimum; maximum) were performed. Categorical variables were reported by percentage (%).

The two-wat random interclass correlation (ICC) analysis was used to evaluate the inter-rater agreement of the classification assigned by the two examiners. Interclass correlation coefficient and 95% CI were reported with a significance level set at 0.05. A 4-level nomenclature was used to assess the level of agreement: poor (0.00–0.50), moderate (0.50–0.75), good (0.75–0.90), excellent agreement (0.90–1.00) [38].

A multinomial logistic regression was conducted to analyze the relationship between the defect type (classification) and various predictor variables related to the peri-implant defects (including age, gender, smoking status, diabetes, jaw and implant locations, submerged time, the history of previous ridge preservation, previous guided bone regeneration, and simultaneous GBR). Predictor variables were input by the forward stepwise model and a relative risk (RR) ratio expressed in exp(B) was reported in each defect type compared to the healthy state of peri-implant bone condition. The statistical analysis was completed by SPSS software (version 29, IBM SPSS Statistics for Mac).

## Results

### Main results

Seventy-four patients attending three private offices in Michigan between the years 2021 and 2023 for implant placement and subsequent uncover stage were included in this study. The patient population consisted of 32 male (mean age 63 years old) and 42 female (mean age 61 years old) patients exhibiting a total of 106 implants. A total of 64, 38, and 4 implants were placed in single tooth gaps, partial edentulous ridges with 2 adjacent missing teeth, and a fully edentulous arch. The distribution of surgeries across different regions was as follows: 12% (n = 13) in the anterosuperior, 6% (n = 6) in the anteroinferior, 40% (n = 42) in the posterosuperior, and 42% (n = 45) in the posteroinferior sectors (Table 2). Additionally, 27% (n = 29) had undergone alveolar ridge preservation at least six months prior to the surgical placement, while 12% (n = 13) had received guided bone regeneration (GBR) beforehand, and 45% (n = 48) were placed with simultaneous GBR.

**Table 2:** Systemic and local characteristics of the included cases

Implant level (n = 106)	Incidence (n)	Incidence (100%)
*Smoking habit*		
Non	89	84%
Current	9	8%
Former	5	5%
No info	3	3%
*Diabetes*		
Current	12	11%
Non	91	86%
No info	3	3%
*Systemic health*		
Other systemic diseases	23	22%
Healthy	42	39%
No info	41	39%
*Surgical site*		
Anterosuperior	13	12%
Anteroinferior	6	6%
Posterosuperior	42	40%
Posteroinferior	45	42%
*Alveolar ridge preservation*		
Yes	29	27%
No	77	73%
*Previous GBR*		
Yes	13	12%
No	93	88%
*Simultaneous GBR*		
Yes	48	45%
No	58	55%

**Fig. 2:**
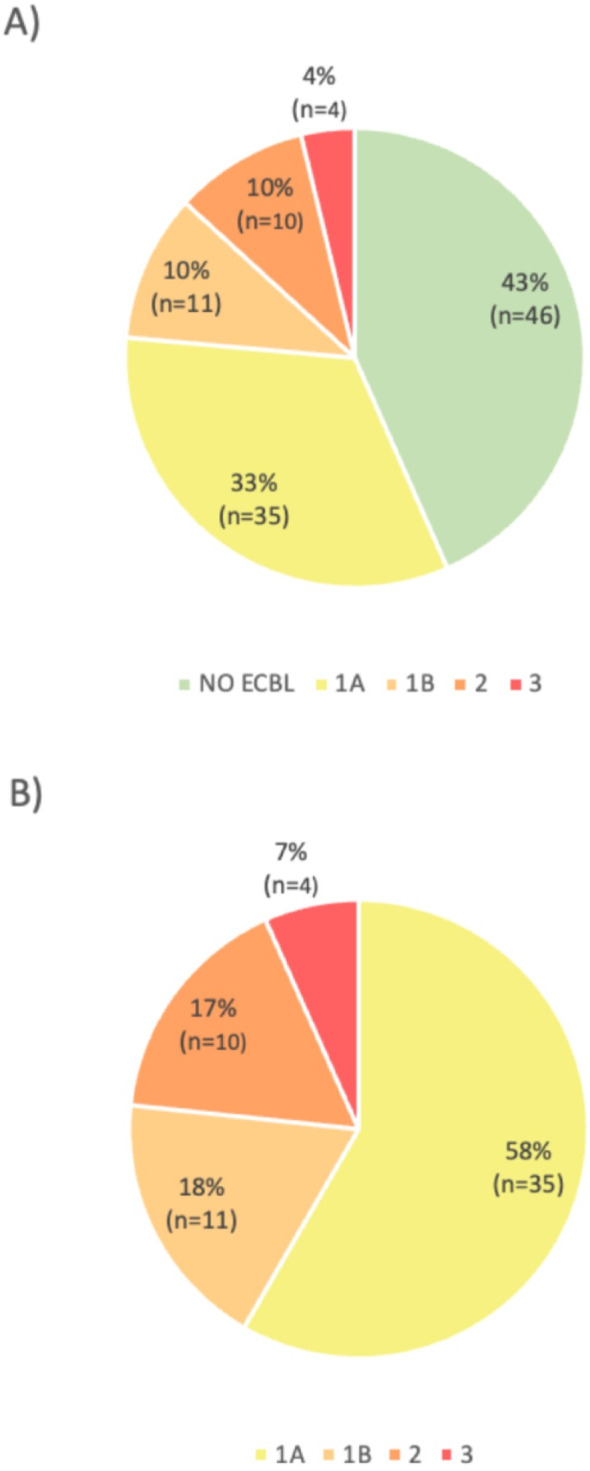
Peri-implant bone loss defect incidence. **A** Total of the implants. **B** Total of the defects

All implants were restored as single crowns, except for the 4 implants for a removable overdenture. The mean healing time from implant placement to the 2nd stage is 177.6 $\pm$ 86.7 days. At the second stage surgery, it was observed that 43% (n = 46) of the implants were covered by bone or did not have any bone loss. Among the remaining 57% (n = 60) of the implants, the distribution of peri-implant bone defect types was as follows: 58% (n = 35) were classified as type 1A, 18% (n = 11) as type 1B, 17% (n = 10) as type 2, and 7% (n = 4) as type 3 (Table 2 and Fig. 2a and b). The interclass correlation coefficient between the two examiners (inter-rater agreement) was calculated for the assignment of peri-implant defect classification. It was 0.85 (0.76–0.90) with significance level < 0.001, which indicated that the inter-rater agreement between two examiners for the peri-implant defect classification was good to excellent.

### Systemic characteristics of the cohort

A significant majority of the implants were placed in non-smoking patients (84%, n = 106), those without diabetes (86%, n = 106), and individuals deemed systemically healthy (39%, n = 106). Conversely, 13% (n = 14) of the population reported current or past smoking habits, 11% (n = 12) were diagnosed with diabetes, and 22% (n = 23) had other systemic conditions, including arthritis, hypertension, various heart diseases, thyroid disorders, cancer, high cholesterol, xerostomia, hepatitis, or bruxism. It is noteworthy that, regarding the overall number of implants placed, information concerning the systemic health status of patients (22%, n = 41), their smoking history (3%, n = 3), and the presence of diabetes (3%, n = 3) could not be acquired from the private office Table 2.

### Implant site characteristics

At the time of implant placement, the mean facial bone thickness measured at the platform was 0.71 $\pm$ 0.76 mm for all cases, including simultaneous GBR, and 1.29 $\pm$ 0.56 mm for cases excluding simultaneous GBR. Most GBR cases were for correcting facial bony dehiscence when the facial bone thickness was recorded as 0 mm. This resulted in the lower thickness reporting when simultaneous GBR cases were included.

Specifically for cases with simultaneous GBR, the facial dehiscence width (mesial-distal distance), length (apico-coronal distance), and facial bone thickness at implant platform were 2.83 $\pm$ 1.21 mm, 2.15 $\pm$ 1.66 mm, and 0.04 $\pm$ 0.19 mm, respectively. At 2nd uncover stage, the corresponding measurements were 1.56 $\pm$ 1.78 mm, 0.8 $\pm$ 1.14 mm, and 0.9 $\pm$ 0.71 mm. In other words, the facial bony defect width and length reduced by 1.26 $\pm$ 1.46 mm and 1.33 $\pm$ 1.78 mm, respectively, with a gain of 0.78 $\pm$ 0.64 mm in facial bone thickness.

The overall model fitting of multinomial logistic regression based on the using a forward stepwise model was statistically significant (*p* < 0.01), indicating that it was able to distinguish effectively between the defect type (classification) based on the predictor variables χ^2^ = 42.04, *p* < 0.001. Among all potential predictor variables, only age, ridge preservation history, and simultaneous GBR were found to be significant with *p* value < 0.001, < 0.001, and 0.034, respectively (Table 3).

**Table 3:** Likelihood Ratio Test were used to determine the impact of the predictors on the goodness of model fit

	Model fitting criteria	Likelihood ratio tests	
	Chi-square	df	Significance
Ridge preservation history	10.45	4	0.034
Simultaneous GBR	18.79	4	< 0.01
Age	19.45	4	< 0.01

df, degree of freedom

For the Type 1A defect compared to the healthy state, the ridge preservation history was the significant predictor. Type 1A defect was more likely to be found on patients without the history of ridge preservation compared to patients with ridge preservation history. The relative risk ratio is 13.3 (95%CI = 1.42–123.9, *p* = 0.02) (Table 4). In other words, the expected odds of exhibiting a Type 1A defect versus healthy is higher for subjects who are without previous ridge preservation. Age and simultaneous GBR were not significant predictors in this comparison.

**Table 4:** Statistically significant predictors in different comparisons to the reference type

Defect type	Reference	Variable	β	*SE*	Odds ratio	95% CI	*p*-value
Type 1A	Healthy	ARP	2.6	1.14	13.3	1.4–123.9	0.02
Type 1B	Healthy	Age	− 0.37	0.17	0.7	0.5–0.95	0.03
Type 2	Healthy	s-GBR	− 3.18	1.54	0.04	0.002–0.8	0.04
Type 1B	Type 1A	ARP	− 7.74	3.43	0	< 0.001–0.4	0.03
Type 1B	Type 1A	s-GBR	24.41	10.7	3.98E + 10	32.3–4.9E + 19	0.02
Type 1B	Type 1A	ARP	− 0.35	0.17	0.71	0.5–0.98	0.04
Type 2	Type 1A	ARP	− 3.74	1.9	0.024	0.001–0.98	0.049
Type 2	Type 1A	s-GBR	10.01	5.03	2.23E + 4	1.2–4.29E + 8	0.047
Type 3	Type 1A	s GBR	− 28.92	10.03	2.76E-13	8E-22–9.4E-05	0.004
Type 1A	Type 3	ARP	− 15.77	1.9	1.10E-08	4.3E-10–2.6E-07	< 0.01
Type 1A	Type 3	s-GBR	28.91	10.21	3.60E + 12	7465.5–1.8E + 21	0.01
Type 1B	Type 3	ARP	− 23.5	3.5	6.20E-11	6.5E-14–5.9E-08	< 0.01
Type 1B	Type 3	s-GBR	53.24	14.47	1.40E + 23	7E + 10–2/98E + 35	< 0.01
Type 2	Type 3	S-GBR	38.93	10.69	8.10E + 16	6.46E + 7–1.0E + 26	< 0.01

ARP, history of alveolar ridge preservation; s-GBR, simultaneous guided bone regeneration; SE, standard error; β, coefficient

When all other types of peri-implant defects were compared to the Type 1A, patients with ridge preservation performed prior to implant placement showed higher likelihood to exhibit Type 1B and 2 defects than patients without, *p* = 0.03 and 0.049, respectively. Similarly, there were higher likelihood of Type 1B and 2 compared to Type 1A on patients with the simultaneous GBR at the time of implant placement than those patients without (*p* = 0.02 and *p* = 0.047, respectively) (Table 4). Reversely, lower likelihood of Type 3 compared to Type 1A on patients with the simultaneous GBR compared to patients without (*p* = 0.004). Interestingly, each year increase in age was associated with a decrease in the likelihood of occurrence of Type 1B defect compared to Type 1A. The relative risk ratio is 0.7 (95%CI = 0.51–0.99), *p* = 0.04.

In the comparison between Type 1B and healthy state, age was the significant predictor. Each year increase in age was associated with a decrease in the likelihood of occurrence of type 1B defect over the healthy state. The relative risk ratio is 0.69 (95%CI = 0.50–0.95), *p* = 0.03 (Table 4).

For the Type 2 defect compared to the healthy state, patients without simultaneous GBR was found with less risk for the occurrence of Type 2 defect (RR ratio = 0.04, 95%CI = 0.002–0.85) (Table 4). In other words, simultaneous GBR increases the relative risk of Type 2 defect compared to healthy state. Age and the history of ridge preservation were not significant in this comparison.

For the Type 3 defect compared to the healthy state, there were no significant predictors found in the comparison. However, in the comparison using Type 3 as reference, the expected risk for Type 1A, 1B, 2 defects were higher than Type 3 when there is simultaneous GBR (*p* < 0.001). Similarly, the risk for Type 1A and 1B defects were higher than Type 3 when there is previous ridge preservation (*p* < 0.001) (Table 4).

## Discussion

The significance of assessing peri-implant bone resorption even prior to the insertion of the healing abutment is under emphasized. A substantial proportion of bone loss (over 60% of total bone resorption) may occur between the second stage and implant loading. This is partially due to bacterial contamination once the implant is exposed to the oral cavity [39]. Bone loss prior to the second stage may also result from surgical factors such as excessive toque, overheating during drilling, implant positioning, or flap elevation [40–42]. Anatomical features like soft tissue thickness [43] and healing-related complications, including cover screw exposure due to soft tissue dehiscence [44], also contribute. Early loss of bone protection could increase the risk of peri-implantitis. Indeed, when marginal bone loss (MBL) exceeds 0.44 mm at six months after loading, progression is significantly more likely, raising the risk of implant failure [33, 45]. Although bone changes alone, may not indicate pathology, such as physiologic remodeling, composite variables such as ≥ 2 mm MBL with bleeding on probing should be regarded as “red flags” requiring clinical evaluation [46].

To the best of the authors’ knowledge, this is the first study that assesses the morphologic features and severity of bone loss defects in 360 degrees at the uncover stage since published literature on this subject primarily concentrates on quantifying the extent of per-implant bone resorption during the uncovering of submerged implants. A systematic review and meta-analysis examined the influence of the implant-abutment junction position on crestal bone loss. The findings indicated that for implants positioned equicrestally, the crestal bone loss (CBL) prior to the connection of the abutment was measured at 0.57 ± 0.29 mm [17]. Conversely, in a study that measures the risk associated with the premature exposure of cover screws between implant placement and uncovering, Hertel et al. reported a bone loss of 0.3 mm (with a range of − 2.4 to 1.1 mm of bone remodeling) around implants during the second stage of surgery; however, they did not specify whether this loss occurred in implants placed supra, equi, or subcrestally. Nevertheless, supracrestal implant position had a higher association with premature exposure (18.3%) which in turn may result in marginal bone loss [44]. Additionally, while the present article does not provide specific measurements for peri-implant bone resorption, it was noted that peri-implant bone loss at the time of the second stage surgery was the most observed phenomenon, occurring in 57% of all uncovered implants. Notably, when analyzing the most prevalent defect, vestibular or lingual/palatal bone loss measuring less than two millimeters was identified as the most frequent type (58% of all defects), corroborating existing literature that indicates bone resorption of less than 2 mm at the time of implant uncovering [44]. All the implants in the present study were placed equicrestally, which could, to a certain degree, reduce marginal bone remodeling by avoiding soft tissue dehiscence throughout the healing phase, thus eliminating this contributing factor.

In a prospective cohort study performed by Cassetta et al. a total of 493 implants were successfully placed utilizing a two-stage surgical approach. The depth of implant placement was determined through random allocation, encompassing crestal, supra-crestal, and sub-crestal positions. Peri-apical radiographs were obtained during the initial implant surgery and again at the second stage two months later. At the two-month follow-up, an average bone loss of 0.86 mm was recorded from the time of implant insertion. Among the implant sites, 175 (35.5%) exhibited bone gain, while 318 (64.5%) experienced bone loss. Notably, three (0.6%) implant sites demonstrated a loss exceeding 2 mm of bone, and two implants (0.4%) experienced a bone loss greater than 3 mm [47]. The findings of our study demonstrated that 57% of implants experienced some degree of bone loss at second-stage surgery, which aligns with the referenced study where 65.5% of implants showed bone loss. However, while the referenced study reported an average bone loss of 0.86 mm and identified cases exceeding 2–3 mm of bone loss (1% of cases), it is constrained by its exclusive focus on mesial and distal measurements, as the review was conducted using x-ray imaging. This method does not provide the capability to assess the presence or absence of bone loss in the vestibular or lingual/palatal regions. Alternatively, our classification system offered a more comprehensive understanding of defect morphology rather than just linear measurements, enabling for future better treatment planning and outcome assessment.

In a retrospective study performed by Vilela et al., the incidence and related factors associated with preloading peri-implant crestal bone loss was assessed. A total of 575 implants in 241 patients were included in the final analysis. The authors observed marginal bone loss of ≥ 0.5 mm in 40.1% (73 out of 314 implants) and a loss of ≥ 1.5 mm in 37.2% (29 out of 358 implants) of equicrestally placed implants [48]. However, no description of the collar smooth/rough surface was made. In this instance, the presence of a rough neck on the analyzed implants could account for the observed reduction in bone loss percentage when compared to the current study, which reported a bone loss rate of 57% (n = 108).

In the present study, we identified various predictive factors that could contribute to early bone remodeling. Regarding alveolar ridge preservation, the occurrence of Type 1A defects was significantly reduced when this surgical procedure was performed, in contrast to pristine sites, demonstrating a relative risk ratio of 13.2. These findings align with those of Marconcini et al., who reported increased marginal bone loss in nongrafted sites. Nevertheless, these results were observed four years after crown delivery [49]. However, these similarities may shed light on the potential progression of bone loss that may occur and suggest that this process could have begun prior to the second stage of surgery, maintaining a consistent loss overtime. Cases with implant placement with simultaneous GBR, were observed to frequently exhibit defects Type 1B and 2. One potential biological explanation for this observation may be that the bone loss resulted from the distance between the native bone and the graft, given that the graft is situated on the avascular surface of the implant, which fails to provide sufficient blood flow and cellular resources [50]. It is expected that there will be some bone loss in implants located in sites where a GBR has been performed (1.40–1.90 mm) over time [51, 52]. Therefore, an early bone defect around implants in GBR sites, even before the healing abutment is placed, may heighten the risk of future bone loss. Interestingly, we did not identify any predictive factors for the type 3 defect, which complicates its prediction.

The aim of these findings and following classification and the predictive factors of bone remodeling in the uncover stage, is to emphasize the different types of defects that can develop even before the connection of a healing abutment or crown installation and subsequent bone remodeling [9]. It is crucial to minimize exposed surfaces of the implant to reduce the risk of future periimplantitis. It has been demonstrated that interproximal exposed threads, such as Type 2 and 3 defects from the present study, may increase the risk of periimplantitis by eight times, with an additional four-fold risk for each extra exposed thread [53]. Additionally, if this bone remodeling continues after the crown is delivered and results in a loss of more than 0.44 mm at 6 months, the rate of marginal bone loss will be significantly higher in the future [33].

### Clinical relevance

Although clinical significance and recommendations cannot be drawn from this study due to its limitations, this study aims to introduce a classification system that may guide future research on peri-implantitis risk and support development of defect-specific treatment protocols. For Type 1A and 1B defects, the presence of bone dehiscence increases the risk of peri-implant recession, making thick peri-implant mucosa essential, when unachievable, a connective tissue graft may be required [54, 55]. In Type 1B defects, diminished potential for interdental papilla formation necessitates close monitoring, and restoration with increased emergence angles may help fill embrasures, though they may rise peri-implantitis risk [56]. Type 2 or circumferential defects carry higher risk of bone loss, requiring continuous monitoring [57]. Some Type 3 defects may ultimately demand explantation, depending on bone resorption extent [4]. Current evidence is limited; therefore, these observations should not serve as treatment directives but rather highlight the need for studies defining effective, defect-specific therapeutic protocols.

### Limitations

A limitation of this study is the undetermined correlation between ridge thickness and defect presence/configuration. Narrower ridges with reduced vestibular bone during implant placement may increase resorption risk [55]. Additionally, the implants used had a 0.5 mm polished collar, which may hinder osteoblast adhesion and contribute to bone resorption [58, 59]. Both factors could influence defect incidence, particularly Type 1A, involving vestibular and/or lingual/palatal surfaces. Thin buccal bone and/or a machined collar may increase these defects. Other limitations are: (1) the diversity of nature of the edentulous ridges, including naturally healing, pre-augmented, and simultaneously augmented, (2) missing information on soft tissue thickness, which could influence bone topography, and (3) potentially unreliable results of the multivariate regression analysis due to small sample size of Type 3 category. Bone remodeling could also be influenced by bucco-lingual implant position and insertion torque. Further research is recommended on the relationship between defect morphology, subsequent bone loss, and peri-implantitis, as well as on developing decision trees for treatment.

## Conclusion

This study proposes a novel classification for early peri-implant bone remodeling at implant uncovering. Findings showed that 57% of implants exhibited some degree of bone loss, with Type 1A being the most frequent (58%). The system offers a standardized framework for assessing early bone loss and may guide targeted treatment and risk assessment strategies. Although further validation is needed, it represents a step toward improving predictability and success in implant therapy.

## Data Availability

No datasets were generated or analysed during the current study.
